# Challenges and opportunities for assisted regional ecosystem adaptation: International experience and implications for adaptation research

**DOI:** 10.1371/journal.pone.0257868

**Published:** 2021-09-24

**Authors:** Karen Vella, Umberto Baresi, Stewart Lockie, Bruce Taylor

**Affiliations:** 1 School of Architecture Built Environment, Queensland University of Technology, Brisbane, Australia; 2 The Cairns Institute, James Cook University, Townsville, Australia; 3 CSIRO, Brisbane, Australia; University of Maryland Baltimore County, UNITED STATES

## Abstract

Maintaining the functional integrity of ecosystems as climate pressures exceed natural rates of adaptation requires new knowledge and new approaches to governance and management. However, research into management interventions to assist regional ecosystem adaptation has generated both scientific and ethical debate. This paper reviews experience to date in order to identify the challenges and opportunities for assisted regional ecosystem adaptation and reflect on the implications for ongoing adaptation research. The review was informed by a database and structured analysis of some 450 reports, peer-reviewed manuscripts and books on participation theory and experience with novel technology development and assisted ecosystem adaptation. We identified five classes of challenges to adaptation research: 1) scientific conflicts and debates over the “facts”, 2) social challenges, 3) governance challenges, 4) epistemic challenges, and 5) ontological conflicts. We argue that engagement strategies linked to the multiple objectives of adaptation research provide opportunities for ecosystem adaptation.

## Introduction

Responding to risks associated with anthropogenic climate change requires mitigation of greenhouse gas emissions to reduce the magnitude and speed of environmental change and adapt to the changes experienced. Though world leaders are moving towards low-carbon economies and experimenting with planetary-scale geoengineering to buy time while de-carbonization strategies are implemented [1, 2], progress has been slow [3]. It is now acknowledged that even under best case scenarios the planet will experience climate extremes that will have catastrophic environmental and human costs [4].

Assisted regional ecosystem adaptation is newly emerging in response to unavoidable climate extremes. As an umbrella concept, assisted regional ecosystem adaptation refers to strategies that could be applied to shift the biogeographic range of species within ecosystems or landscape management and technology solutions that can be applied to address extreme climate effects at the regional scale [5–7]. Types of assisted regional ecosystem adaptation strategies include the restoration of key sites within ecosystems [5, 7], regional geoengineering to moderate the effects of climate change and provide time for ecosystems to adjust to external change (thus ‘buying time’ for evolutionary responses) [6], and assisted evolutionary responses such as assisted gene flow, selective breeding, or other means [5]. These strategies include both restoration elements and strategies that assist ecosystems to cope with and adapt to change. This is distinct from global geoengineering technologies applied at the planetary scale to manipulate the earth’s climate and counteract climate change [8]. Assisted regional ecosystem adaptation is also distinct from passive restoration, which involves natural recovery following the removal of contaminative sources (e.g. carbon emissions) [9].

Regional climate extremes place communities, societies, and regional ecosystems under extreme pressure. Communities, societies and ecosystems already face adaptation pressures that differ from and/or are greater than those experienced historically [10, 11]. Maintaining values such as the productivity of agricultural systems, cultural heritage of landscapes, livability of human settlements and functional integrity of ecosystems will be difficult in the face of climate extremes unless new adaptive capacities are developed. This will require new knowledge, and in all likelihood, new approaches to governance and management. Adaptation research that can identify approaches that can be implemented over the medium- to long-term can assist communities, societies and regional ecosystems to manage change and continuity in the face of climate extremes.

Experience with ecosystem adaptation to date suggests considerable potential over the scientific and ethical merits of intervention [12]. Though engaging with impacted and interested stakeholders is often proposed as a means to minimize conflict over environmental management strategies [13], the heightened complexity of ecosystem adaptation approaches to mitigate extreme climate events requires careful consideration. Understanding the challenges that underlie adaptation research is a first step in creating meaningful opportunities for participation in decision-making [14].

This paper considers the challenges associated with assisted regional ecosystem restoration and adaptation (also referred to as ecosystem adaptation) to inform intervention research within the marine ecosystem of Australia’s Great Barrier Reef. The motivation for exploring challenges to assisted ecosystem adaptation research in more depth lies in shaping the Reef Restoration and Adaptation Program, a research and development initiative oriented toward developing adaptation interventions that can be deployed at the scale of Australia’s Great Barrier Reef (GBR). The program’s aim of preventing and repairing ecosystem degradation arising from climate change and other stressors along the 2300 kilometer length of the GBR represents a considerable technical and scientific challenge. It also represents a considerable social, economic and political challenge. While assisted regional ecosystem adaptation in the GBR aims to maintain or enhance ecological and social benefits in the face of environmental change, the risks and benefits may vary substantially across the GBR, its land-based catchments, and the various place-based and interest-based communities that rely on diverse coastal and marine ecosystems to meet a range of social, economic and cultural needs [15, 16]. Given the complexity of the GBR, difficulties in taking action to address greenhouse gas emissions change and challenges of anticipatory climate adaptation, it is reasonable to expect that research into regional ecosystem adaptation will raise public and stakeholder concern and potentially lead to skepticism over the objectives of adaptation research. In 2017, the statutory authority with management and regulatory responsibilities for the GBR, the Great Barrier Reef Marine Park Authority (GBRMPA), signaled the need to accelerate active restoration and resilience-building of the GBR’s coral ecosystems in response to successive mass beaching events and cyclone damage at a summit of partners and stakeholders [17]. This included calls to scale-up local restoration methods and for research and development for large-scale restoration methods [17]. Within the following three years the GBRMPA had developed new guidelines to support permitting in-water activities, and a subsequent *Policy on Great Barrier Reef Interventions* to guide managers in their objectives:

“to enable restoration and/or adaptation interventions designed to directly support and build ecosystem resilience and provide conservation benefits, at a range of scales, now or in the future to the Great Barrier Reef” [18, p.1 section 3a].

The aims of this paper are: 1) to identify challenges to ecosystem adaptation and 2) reflect on the implications and opportunities for adaptation research in the GBR. To achieve this, a conceptual framework of the challenges to assisted ecosystem adaptation drawing on a database of 450 reports, peer-reviewed manuscripts and books was developed. The following sections outline the methods, results, discussion and conclusion.

## Methods

The analysis was based on a database and structured analysis of publications concerned with political, social, technical, and technological dimensions of assisted regional ecosystem restoration and adaptation. The database was built and analyzed between May and September 2018. Publications relevant to the structured literature review were identified through keyword-based database searches in the online ‘Elsevier Scopus’ research portal. These keywords were organized against five provisional themes, including risk assessment, public participation, restoration and technology, restoration case studies, and assisted adaptation. These keywords were identified by the authors through brainstorming sessions. These brainstorming sessions built upon the authors’ experience and knowledge in the fields of GBR, ecosystem adaptation and restoration, stakeholder identification and engagement, governance, risk management and technology deployment in the GBR. The search in ‘Elsevier Scopus’ was initially performed by a research assistant, a co-author of this paper, and then validated by all authors. Keywords were mapped for five areas of analysis:

1. **Risk management** (78 documents). Keywords: "risk governance" & "social framework"; "ecological restoration" & "risk governance"; "ecological restoration" & "risk" & "governance" & "marine"; "ecological restoration" & "risk" & "governance" & "reef"; "ecological restoration" & "risk evaluation"; "ecological restoration" & "risk analysis"* &"marine"; "ecological restoration" & "risk analysis" & "reef"; "risk governance" & "ecological restoration";

2. **Participation** (56 documents). Keywords: "ecological restoration" & "public participation" & "marine"; "ecological restoration" & "public participation" & "reef"; "ecological restoration" & "public engagement"; "ecological restoration" & "public engagement" & "marine"; "ecological restoration" & "public involvement";

3. **Technology** (38 documents). Keywords: "ecological restoration" & "technology innovation"; "ecological restoration" & "public involvement" & "technology"; "ecological restoration" & "public participation" & "technology";

4. **Geoengineering** (112 documents). Keywords: "Large-Scale Geoengineering"; "Geoengineering" & "reef"; "Geoengineering" & habitat restoration"; "major habitat" & "restoration" & "marine" & "reef"; "aggressive environmental intervention"; "environmental intervention" & "Geoengineering"; "environmental intervention" & "rehabilitation" & "marine"; "environmental rehabilitation" & "structural" & "engineering"; "large-scale" & "environmental rehabilitation”; "large-scale environmental intervention"; "environmental rehabilitation" & "marine"; "environmental rehabilitation" & "reef"; "scenario planning" & "ecological" & "restoration"; "everglades" & "governance" (2015–2018); "everglades" & "participation" (2015–2018).

5. **Assisted adaptation** (222 documents). Keywords: “assisted adaptation” & “ecosystem”; “assisted adaptation” & “risk”; “assisted colonization” & “ecosystem” & “marine”; “assisted colonization” & “ecosystem” & “reef”; “assisted colonization” & “ecosystem” & “coral”; “assisted colonization” & “risk” & “marine”; “assisted colonization” & “risk” & “reef”; “assisted colonization” & “risk” & “coral”; “assisted migration” & “ecosystem” & “marine”; “assisted migration” & “ecosystem” & “reef”; “assisted migration” & “ecosystem” & “coral”; “assisted migration” & “coral”; “assisted migration” & “ecosystem” & “risk” & “marine”; “assisted migration” & “ecosystem” & “risk” & “reef”; “assisted migration” & “ecosystem” & “risk” & “coral”.

The first keyword was searched in the document titles, keywords and abstracts. The other keywords were searched in the full text. The search included all peer-reviewed documents published in English (journal articles, conference proceedings, books, and reports). All years of publications were considered (1979–2018), with the first document published in 1979. The search identified 506 relevant publications. Following the removal of duplicates and inaccessible publications, 347 publications were available for analysis. Consultation with a broader reference group of professionals and scholars contributed an additional 138 publications. Overall, 485 papers were included in the database and structured literature review. Several of the documents analyzed covered more than one challenge. To avoid repetition and to keep references meaningful and strictly adherent to each concept, Table 1 identifies the most relevant reference. At times, the literature review presents only one meaningful reference for each challenge. This is due to the unevenness of the literature on these topics rather than a lack of relevance of these challenges for assisted ecosystem adaptation research.

**Table 1:** Challenges to adaptation research.

Group	Themes From Literature	Details	References
1. Scientific debate (the “facts”)	a. Complexity of assisted adaptation/restoration	Many natural factors are affected (landscape, climate, and vegetation communities)	[19]
		Many stakeholders affected by new technology	[20]
		Conflicting approaches (e.g. ‘agronomic approach’ versus ‘ecological approach’)	[7]
	b. Practicality, cost-effectiveness and timeliness of intervention at scale	Cost-effective geographic priorities for intervention	[21]
		Known techniques seem more cost-effective than new technology	[22]
		Passive restoration appears more cost-effective than active restoration	[9]
		Restoration activities depend on many disciplines	[7]
	c. Complexity of geoengineering interventions (from global to regional levels)	Uncertainty for human-ecosystem equilibrium of solar radiation management (SRM)	[23]
2. Social	a. Lack of awareness or understanding of the phenomena at stake	Understanding shapes human attitudes	[24]
		Misinterpretation of information, illusionary correlation, selective information scrutiny, information-processing problems, political affiliation and cognitive function	[25]
		Understanding is necessary but insufficient to achieve consensus	[26]
	b. Risk perceptions can be amplified or attenuated by more information.	Difficult achievement of social acceptance on new technology without risk assessment and management	[27]
		Personal and institutional control, voluntariness, familiarity, dread, inequitable distribution of risks and benefits, artificiality of risk source, and blame	[28]
		Complexity of risk damage, risk probability, and social mobilization	[29]
	c. Distrust of scientific and management institutions	Trust in institutions necessary to accept new technology with unknown risks	[30]
		Risk-concerns induced social conflict undermines trust in policy and science	[31]
		Lack of trust in state-backed projects	[32]
	d. Moral hazards with SRM; technology used to justify inaction on mitigation	SRM geoengineering as a quick-fix to keep ignoring causes of environmental degradation	[33]
		SRM geoengineering interventions as ‘techno-fixes’ to neglect mitigation efforts	[34]
	e. Global geoengineering: stratospheric aerosol injection (SAI) and carbon capture and storage at a planetary scale.	Public tensions and dilemmas for ambiguity on SAI geoengineering	[8]
		State of nature, control over nature, trust in institution, altruism, egoism, risk aversion around SAI	[35]
		Technology acceptance depends on trust in scientists and firms	[36]
		SAI technical information’s unpredictable effect on the public.	[37]
		SAI geoengineering as a ‘sign of surrender’ or a ‘panic action’	
		Distrust in institutions if new technology on carbon capture and storage helps in shirking responsibilities for climate change	[38]
3. Governance	a. Transboundary responsibilities and impacts (political, jurisdictional and sectoral) of new technology	Carbon capture interventions as ‘techno-fixes’ to neglect mitigation efforts	[34]
		Lack of clarity in legislative frameworks	[39]
	b. Lack of accountability for unintended consequences	Need of schemes for accountability	[40]
		Framing of scientific and social responsibility on research and innovation	[41]
	c. Global transboundary management of geoengineering technology	Governance problems from transboundary effects of geoengineering	[42]
		Risk of unregulated, unilateral, or self-interested uses	[43]
		Openness versus secrecy of field experimentations–public engagement versus deference	[8]
4. Epistemic	a. Managing uncertainties due to spatial and temporal distance between interventions and effects	Long-time intervention effects versus slow dynamics in play	[44]
		Resolving past conflicts versus discovering sustainable futures	[45]
		Short term ‘safety’ versus long term ‘sustainability’	[46]
	b. Authority. Scientific expertise vis-à-vis lay, community, Indigenous perspectives	Public engagement only on ethics and values, not on scientific expertise	[26]
		Skepticism, scandals and controversies, and troubled alignment with indigenous epistemologies and representation	[47]
		Information and communication gaps between scientists and practitioners	[48]
		Problematic meetings often degraded to simple information moments	[49]
5. Ontological conflict	a. Confronting the ‘naturalness’ or ‘artifice’ of intervention	‘Pleasure-related’ versus ‘responsibility-related’ emotional motivations to protect nature	[50]
	b. Defining the goals of intervention	Environmental restoration versus ecological restoration	[51]
		Complex choice of conservation targets (role of values and cultural commitments)	[52]
	c. Ethics of intervention	Ethical divisions on protecting nature against individual freedom	[53]
	d. Geoengineering at the planetary scale	Perceived ‘naturalness’ of occasional phenomena versus ‘artificiality’ of human-induced changes	[42]
		Geoengineering perceived as too divergent from natural processes	[54]
		Geoengineering implications: threat to nature, security for future generations, unexpected consequences, unsustainable lifestyle, ‘natural’ geoengineering	[55]
		Side effects: ocean acidification, alter precipitation patterns, unilateral deployment, and abrupt halting	[56]
		Defining responsibility and eligibility for compensation, and compensation itself	[57]
		Political destabilization and conflict for impacts or fears of geoengineering used for immoral purposes	[43]

The analyzed publications were considered suitable depending on their relevance to the GBR case, and more precisely, to the range of interventions that Reef Restoration and Adaptation Program may deploy at a regional level. The case study involved the investigation of solar radiation management at the regional level. Though global geoengineering interventions raise social, ethical, and technical problems not relevant to the scale of our study, some of this literature may inform the types of challenges required for assisted regional ecosystem adaptation. We acknowledge that the scale of impact may differ; however, this differentiation may not always be clear to communities or stakeholders, and global efforts could potentially raise concerns that regional efforts need to consider. Literature associated with global geoengineering was therefore included in our analysis; however, the findings are separated in the results.

The 42 documents significant to identifying challenges to assisted regional ecosystem adaptation are described in a S1 Table. This table identifies the types of journals and geographies for each document. The majority (60%) were empirical in nature and focused on a discussion of original findings linked to the collection of new data. Most documents focused on European countries (26%) and North America (24%), with few cases studies located in Africa (2), Asia (2), and Oceania (1).

Using the ScimagoJr online portal (https://www.scimagojr.com/), we identified the subject areas of the journals publishing these 42 documents. This method was not without limitations, considering that most journal are classified in several subject areas. However, it helped to understand how publications were distributed in journals associated with different areas. We found that most publications were in journals related to environmental science (34%), social sciences (18%), Earth and planetary sciences (12%), and agricultural and biological sciences (10%). While journals focusing on environmental science and agricultural and biological sciences presented mostly empirical research (70%), journals focusing on social sciences and Earth and planetary sciences presented equal distributions between empirical studies and conceptual documents. The most represented journals were climatic change (four documents), bioscience (three documents), and restoration ecology (three documents). Of the 42 documents, 13 related to geoengineering. As previously mentioned, we considered these documents in a separate category in the results because the scale of interventions, governance needs and community of stakeholders affected have implications on challenges for research.

## Results: Challenges to assisted ecosystem adaptation research

Experiences of assisted regional ecosystem adaptation highlighted several potential points of conflict related to technical considerations embedded in scientific debates, the nature and potential risks of technology deployment, and social and political issues related to new technology. These were classified these into five groups of challenges: 1) scientific debate, 2) social, 3) governance, 4) epistemic, and 5) ontological conflict. These are summarized in Table 1 and outlined below. The findings from the global geoengineering literature relating to carbon capture and storage and stratospheric aerosol injection are separately identified. Solar radiation management, which can be applied at regional scales, is also presented separately.

### Scientific debate

Assisted adaptation and restoration projects that require deployment of new technology often involve a high degree of complexity, which can trigger high degrees of uncertainty, confusion and emotional detachment and distrust amongst the general public [31]. Avoiding the use of ambivalent linguistic frames and instead considering how the public perceives technical terms and concepts can help reduce detachment and distrust [58]. However, there is more at stake here than how complexity is communicated.

Complexity here has several dimensions (Challenge 1a: Complexity of assisted adaptation/restoration). Novel adaptation or restoration interventions influence interrelationships between a plethora of ecosystem features and processes, including landscape characteristics, climate, and biological communities, with the potential for unexpected outcomes [19]. For example, China’s ongoing large-scale ecological restoration practices show how unharmonized modifications of natural factors can trigger restoration difficulties and side effects that harm both nature and society [19]. The complexity of new technology demands that a high number and many types of stakeholders be engaged [20], yet bringing diverse interests and perspectives together is associated with its own potential for unintended or undesirable outcomes, including conflict over objectives. For example, the restoration of degraded agricultural land has been characterized by diverging perspectives regarding the desirability of an ‘agronomic approach’ focused on agricultural productivity versus an ‘ecological approach’ focused on ecosystem functionality [7].

The need to decide which technology to use, for which purposes, and how calls for reflections–and consequent dilemmas–on practicality and cost-effectiveness (Challenge 1b: Practicality, cost-effectiveness, and timeliness of intervention at scale). Before deploying new technology on a large scale, small-scale tests and geographic priorities–or hotspots–for intervention need to be ascertained [21]. Effective estimations of the cost-effectiveness of interventions in priority hotspots require contributions from several disciplines, as shown by the restoration of agricultural landscapes in the Lower Murray region of South-Eastern Australia [21]. To achieve this, knowledge from several disciplines needs to be considered to shape suitable and effective interventions using what is referred to in ecological restoration as a ‘restoration toolbox’ [7]. However, due to the inherent complexity of new technology and its deployment, technology that is better known and understood is commonly viewed as preferable and more cost-effective [22]. Options granting the least amount of intervention required are often perceived as preferable. An example is passive restoration, which has been perceived as more practical and cost-effective, and thus more appealing than active restoration [9].

### Social challenges

Social factors relate to knowledge, risk perception, relationship with institutions, and how technology-based approaches for ecosystem adaptation are framed within broader climate agendas (Challenge group 2: Social challenges). These factors can exacerbate problems associated with a lack of awareness and understanding by the public (Challenge 2a: Lack of awareness or understanding). Attitudes towards ecological adaptation and restoration vary depending on the understanding of the management initiative [24]. The salt marsh restoration project on Martha’s Vineyard, Massachusetts shows the importance of building a shared understanding of the management initiative, and targeting the different resource user-groups to identify, prioritize, and quickly act upon areas of agreement [24]. The idea that “once the public understands the ‘real’ issues, then it will trust institutions, a ‘reasonable’ consensus will arise, and policy-making can proceed” is deceptive [26, p.175]. Providing information on new technology does not necessarily translate into consensus around technology use, as changes of attitude towards new technology are complicated by factors such as misinterpreting information, selectively scrutinizing information, and political affiliation [25].

Informing and engaging with the public to create consensus around new technology requires addressing people’s concerns about the risks that technology could bring to their everyday lives (Challenge 2b: Risk perception). The literature on global geoengineering has demonstrated that providing more information, or providing information alone does not necessarily promote consensus [8, 33, 35, 42, 59]. The literature on ecosystem restoration demonstrates that people need to be involved in risk assessment and management frameworks to prevent or dissipate suspicion around new technology [27]. This should not only clearly identify risk features such as risk damage, type of risk probability, and type of social mobilization [29], it should also consider the factors shaping individual perception such as personal control, institutional control, voluntariness, familiarity, dread, inequitable distribution of risks and benefits, artificiality of risk source, and blame [28].

Trust in scientific and management institutions (Challenge 2c: Distrust of scientific and management institutions) is a key factor in determining the success or failure of acceptance of new technology. When declining trust generates social conflict, it obstructs technological development and can further reduce trust in science, scientific institutions, and science policy [31]. The performance of institutions and public authorities is essential for trust and engagement. Poor performance severely reduces stakeholders’ trust in state-backed projects [32]. The attempted restoration of depleted cereal fallows in arid Tunisia showcases the possible causes of community distrust in scientific and management institutions. A lack of commitment towards the state-backed project came from conflicting views on crops (some of which were considered sacred by some community groups), divergent beliefs (widespread opinions in contrast to scientific evidence) and from agropastoralists’ lack of trust in state efforts to combat desertification [32]. When trust in institutions is high, the public is more willing to follow their lead on unknown new technology in which risks cannot be adequately estimated [30]. Similarly, experience with global geoengineering found that trust in scientists and management institutions can increase technology acceptance [36], though this can change if the political process fails to adequately address publicly perceived risks or if technology is perceived as a shortcut to addressing climate change [38].

### Governance challenges

Challenges to assisted ecosystem adaptation research and deployment also arise from governance factors associated with legal and political systems. These factors relate to geopolitical and jurisdictional boundaries, sectoral boundaries, divergent community and business interests, and the poor definition of responsibilities for managing unintended consequences associated with new technology (Challenge group 3: Governance challenges).

Transboundary governance problems related to new technology, its regulation, and management of impacts is an issue for geoengineering at the planetary scale [8, 42, 43]. However, the transboundary effects of interventions applied to regional ecosystem adaptations also cause governance problems. Concerns include how interventions will be managed, transparency around management decisions, and public perceptions about the impacts of technology deployment on different communities and how these will be managed to be fair and equitable (Challenge 3a: Transboundary responsibilities and impacts). To improve governance, legislative frameworks must be clear, comprehensible and workable–conditions referred to as ‘institutional tractability’ [39]. However, current legislative frameworks (e.g. European Union) are not suited to managing aspects of ecological adaptation based on the deployment of new technology [39]. New technology creates problems for institutional tractability, jurisdictional rights and responsibilities [39], especially when the management of technology use or ecosystem management crosses multiple political and bureaucratic boundaries. This could be the case of the GBR, where multiple levels of decision-making are linked to several administrative bodies. In such a diversified and complex context, regulation and arrangements may be flawed through: a) lacking a clear univocal terminology, when different bodies use different terms to refer to same elements; b) lacking clear statutory goals that channel efforts towards specific outcomes; c) lacking clear tools and procedures legally enforceable by agencies; and d) lacking governance tools to incentivize, discipline and coordinate numerous actors over large areas [39].

If clear legislative or governance frameworks are not in place, defining the accountability for potential effects of testing or deploying new technology becomes a major cause of public concern (Challenge 3b: Lack of accountability for unintended consequences). Although designing schemes for accountability reduces risk perception [40], this can be complicated by decisions about whether and how to make responsibilities known to the general public. Openness and public engagement favor connections between science and society, helping the community to understand and clearly attribute responsibility to researchers and scientists–preventing lack of accountability and following distrust in scientists [41]. For example, the European Union has recently been working on the concept of ‘responsible research and innovation’, aiming to overcome existing challenges linked to the definition of responsibilities, not only for scientists, but for all categories of stakeholders involved in these processes (universities, innovators, businesses, policy-makers and research funders) [41]. Ultimately, this will improve trust in the process, the authorities involved and the potential outcomes.

### Epistemic challenges

Epistemic challenges arise from a lack of consensus over how valid and reliable knowledge of assisted adaptation can be produced in the absence of certainty about the spatial and temporal dynamics of environmental change and the impact of change on management interventions that have neither been developed nor deployed (Challenge group 4: Epistemic challenges). The experiences and perspectives of a diverse range of stakeholders are implicated in epistemic challenges, including Indigenous peoples and natural resource users [60]. Indigenous groups have often suffered due to green initiatives, suffering what Vanclay described as economic displacement, physical displacement, livelihood impacts, impoverishment, disruption to everyday life and to ecosystem services, and human rights [61]. Local knowledge needs to be considered as important as scientific knowledge, both of which need to properly interact and contribute equally to decision-making [62].

Due to the nature of assisted adaptation and restoration through new technology, it is likely that some uncertainty will not be resolved until deployment takes place. Though simulations are possible, the lack of previous testing in exactly the contexts at stake make accurate and exhaustive predictions impossible (Challenge 4a: Managing uncertainties due to spatial and temporal distance between interventions and effects). The dichotomy between the short term ‘safety’ of interventions with long term ‘sustainability’ horizons means that the intra-generational and inter-generational aspects of risks due to technology deployment need to be properly considered [46]. The slow time horizons of intervention effects (which, as highlighted in the case of the Florida Everglades, can be decades) and slow dynamics in play–whether these are technical, social, or political–create further challenges for engagement in research and decisions about assisted adaptation [44]. As observed in the Florida Everglades restoration project (USA), the naturally slow ecosystem dynamics set a time horizon for the effect and assessment of several decades and complicated decision-making [44]. The focus of governance in the face of high uncertainty and unforeseeable outcomes needs to be on the present and the future, rather than focusing on resolving past conflicts. Focusing on past conflicts be tempting but detrimental for assisted adaptation through new technology [45].

The extended timeframe intrinsic in assisted adaptation and in the deployment of new technology is better addressed when scientific expertise is well integrated in the decision-making process, together with community and less represented perspectives (e.g. indigenous groups) (Challenge 4b: Authority. Scientific expertise vis-à-vis lay, community, Indigenous perspectives). Evidence shows how deliberations about and the implementation of adaptation and restoration are impeded by information and communication gaps between scientists and practitioners [48]. This is often the consequence of polarized ‘scientific’ and ‘public’ positions that lead to engagement and participatory moments more focused on ethics and values rather than on exposing scientific expertise to the scrutiny of the broader community [26]. Meetings with stakeholders or the public that do not delve into scientific details often occur so as to not incur major setbacks with potentially contrary public feedback [26]. Similarly, meetings designed to resolve controversies are often degraded to moments of public information when conflict proves difficult to overcome [49]. This cannot be a solution when consensus needs to be built around the use of new technology. Such behaviors only undermine the confidence of the public and stakeholders in environmental decision-making leading to increased skepticism, controversies, and questions about how different voices and views can actually be heard (e.g. indigenous epistemologies) [47]. Public skepticism and lack of trust can derail participatory processes aiming to increase consensus around restoration projects. These struggles in gaining consensus affect restoration regulation (e.g. the Shale Gas in Kent County, New Brunswick, Canada) [47] and restoration processes (e.g. the Florida Everglades) [48]. Frameworks for collective learning and for the definition of shared values through deliberative processes present a viable solution to overcoming these issues [63, 64].

### Ontological conflict

Ontological conflicts involve competing ideas about the authentic nature of ecosystems and thus the appropriate scope of human intervention (Challenge group 5: Ontological conflict). This is an issue for geoengineering interventions for assisted ecosystem adaptation at both global and regional scales. Ontological conflict has the potential to provoke disagreement over the ethics of assisted adaptation research and intervention. At sub-global levels, ontological conflict is reflected in debates over whether intervention goals ought to be protecting ecosystems from human influence or managing land and seascapes to enhance both ecological and cultural values [65].

When not properly addressed, views on the ‘naturalness’ or ‘artifice’ of interventions on wilderness and natural heritage can generate severe problems for engagement in and acceptance of assisted adaptation and restoration (Challenge 5a: Confronting ‘naturalness’ or ‘artifice’ of intervention). The human desire to see nature achieving specific goals (e.g. species prosperity) has been identified with specific ‘pleasure-related’ goals, while ‘responsibility-related’ goals tend to outline the human drive to avoid undesired outcomes in natural systems (e.g. pollution increase) [50]. Different views can therefore trigger different community, stakeholder and societal goals in ecosystem adaptation (the priority can either be to restore the environmental equilibrium pre-human intervention or to restore selected elements that can be enjoyed by humans).

Defining the goals of intervening to facilitate ecosystem adaptation is paramount because different goals can lead to different interventions and outcomes (Challenge 5b: Defining the goals of intervention). For example, environmental restoration can differ considerably from ecological restoration. The former is focused on remediation and removal of hazardous wastes, while the latter aims to repair damaged ecosystems and enhance their productivity and/or biodiversity [51]. This is the case of areas contaminated during the production of Cold War nuclear weapons in the US, where different goals (e.g. environmental versus ecological restoration) require different interventions, efforts and commitments [51] The choice of conservation targets (e.g., populations, species, habitats, or ecological processes) is a complex process shaped by values and other cultural commitments [52]. Goal selection needs to take risk perceptions and inclusive decision-making processes into consideration.

Many of the questions and open issues presented in this section have ethical implications that stress the need to engage with the public and stakeholders in a two-way process of mutual learning (Challenge 5c: Ethics of intervention). Ethics dilemmas associated with human interventions are also evident when new technology cannot be deployed in a geographically contained area [52], triggering transboundary challenges. Dilemmas regarding the extent of human interventions on the ecosystem include ethical divisions rising from diverse societal and individual commitment to protect nature, among which is the ‘freedom to exploit nature’ [53].

Literature relating to geoengineering suggests that building consensus around deploying new technology requires addressing five areas of public perception: 1) how the intervention preserves or threatens natural systems, 2) how it affects security for future generations, 3) the level of potential unexpected consequences, 4) how radical the geoengineering intervention will be, and 5) how this relates to ‘natural’ forms of geoengineering [55].

## Discussion

Assisted ecosystem adaptation raises questions about the prioritization of research efforts, the management of risk and uncertainty, and the ethics of anticipatory intervention in complex and dynamic systems upon which multiple communities depend for social, cultural, and economic wellbeing. Such questions are not simply matters of fact, they are matters of value, authority and compromise [66]. This review found five factors associated with assisted ecosystem adaptation: 1) scientific debate; 2) social challenges; 3) governance challenges; 4) epistemic challenges; and 5) ontological conflict, provide challenges for deliberation, negotiation and compromise. This is especially challenging in the large, socially and ecologically complex and highly contested GBR region. Simplistic institutional and scientific approaches to action have proven ineffective and disruptive for negotiating consensus and compromise in the past [67]. Though there are also many examples in which consensus and compromise have been negotiated within the GBR [Vella and Baresi, 2017], the complexity and uncertainty associated with assisted ecosystem adaptation further adds to the challenge.

### Scientific debate

Scientific debate over the feasibility and ethics of assisted adaptation illustrates that the scientific community (like others) is heterogeneous and must be meaningfully engaged itself if it is to be mobilized in support of assisted adaptation research [68]. Absolute consensus among scientists is neither practical nor desirable given the dependence of scientific method on principles of falsification and doubt on the one hand, and the value of approaching adaptation challenges from multiple perspectives on the other [69]. Nonetheless, conflict among scientists in the GBR over the merits of devoting research effort to assisted adaptation can add challenges for adaptation research in several important ways. It can undermine public confidence in assisted adaptation research, dis-incentivize scientists from participating in adaptation research, discourage public and private investment in research, and perversely, encourage experimentation with adaptation interventions that are neither informed by nor evaluated with appropriate scientific rigor.

None of the documents included in this review discussed systematic attempts to engage with scientists as a distinct stakeholder group. However, the problem of climate change has already led to substantial innovation in the conduct and organization of science (for example, the institutionalization of a consensus-seeking approach to scientific knowledge synthesis in the Inter-Governmental Panel on Climate Change) [70]. Since the early 2000s, successive federal governments have engaged scientists in consensus-based approaches in addressing water quality problems in the GBR [71]. This suggests that further innovation to support effective adaptation research and practice in the GBR is possible.

### Social challenges and opportunities

Knowledge and understanding, risk perception, trust in scientific and management institutions, and perceptions of how proposed interventions fit within the broader context of climate policy and action provide challenges, and potentially more important, opportunities for adaptation research. It is essential to prepare communities for the intense change required by adaptation and restoration projects, as proven in the cases of the Florida Everglades and the Swedish Kristianstads Vattenrike [44]. The literature review also suggests that the development of decision-making arrangements that are comprehensible to the public and stakeholders is critical to their transparency and to the development or maintenance of trust [72]. Public trust in the process is often related to clear and reliable leadership [72]. Suspicion that adaptation may be pursued as an excuse for ineffective policy and investment to address climate change has been shown to fuel public distrust–distrust that is likely to spill over into concern over the risks and costs of adaptation interventions, skepticism as to their benefits, and withholding of political support [73]. Engagement focused solely on transferring knowledge about adaptation research has similarly been shown to amplify distrust and risk aversion [74]. The logical inference here is not that information provision via social marketing and education campaigns should be curtailed, rather that information provision should be undertaken in concert with participatory engagement strategies given the uncertainties, trade-offs and potential value conflicts that characterize assisted adaptation research.

There is no denying the extra challenge to communication and public participation posed by the highly prospective nature of assisted adaptation research over a system as large and institutionally complex as the GBR. It covers an area of approximately 344,000 square kilometers and is home to a rich variety of Indigenous traditional owners, other communities and stakeholders, and local, state and national governments [75–78]. International agreements concerning World Heritage, the rights of Indigenous peoples’, climate change and biodiversity must all be considered alongside national and state regulatory frameworks and a vast array of relevant social, environmental and industry policy.

In practice, communication and participation are most effective when based on detailed understanding of the relevant social environment, including the full diversity of stakeholders; innovative and flexible methods of information exchange; open and inclusive processes; building shared knowledge as soon as possible; managing power dynamics and giving everyone similar influence and attention; and allowing time for values, aspirations and priorities to change [79]. The involvement of a wider and more diversified set of stakeholders and community groups brings equally diversified positions, beliefs and value systems into ecosystem adaptation conversations, creating challenges for decision making about research and policy programs.

In practice, structuring public and stakeholder engagement in decision-making over such a large area and in context of a highly complex socially institutional and regulatory environment is also technically challenging due to the diverse range of parties and rights holders who have interests at stake. Experiences involving policy-makers in science and community and multistakeholder parties together in dialogic spaces in the GBR [80] are a starting point that can be built upon. Further research into frameworks for supporting diversified involvement might usefully focus on maximizing the effectiveness of adaptive restoration initiatives and minimizing the chances that decisions are made unilaterally by groups of highly skilled but unrepresentative individuals and organizations. This is particularly relevant to the GBR, considering that tensions between scientists and policy-makers have arisen in the past as to whether “scientists should independently (I4) [sic] make policy decisions to set targets and allocate permits” [80, p. 497].

### Governance frameworks

Even though the GBR is a sub-national region, problems around transboundary responsibilities and impacts, lack of clarity for responsibility, and accountability frameworks for regulating new technology are highly relevant across the multiple spatial, social and institutional boundaries relevant to governance in the GBR. Given the many potential challenges discussed above and their implications, there are important questions about the extent to which reef managers–with authority over restoration and adaptation in ecosystems such as the GBR–and the research and development providers engaged in developing and testing novel interventions can practically operationalize these considerations. Early signs from the GBR indicate that while challenging, progress is possible. The GBRMPA’s *Policy on Interventions in the Great Barrier Reef* [18] engages with a number of these concerns, particularly around governance challenges, transparency, and engagement in decision-making and in the assessment of risk.

The GBRMPA Interventions Policy establishes principles to guide decision-making under conditions of uncertainty and evolving scientific and community understandings of the impacts of action or in-action (e.g. sections 43, 44, and 70, GBRMPA Policy on Great Barrier Reef Interventions). Importantly, the policy is explicit about the types of interventions that may be permissible with careful management and oversight, that account for Traditional Owner and community aspirations, and above all, protect the ecological, social, economic and biocultural values of the Marine Park. However, due to the ‘very poor outlook’ for the reef and as knowledge builds on risks and cumulative impacts, the policy also provides scope to revisit and reappraise the suite of currently identified high risk, unacceptable interventions in the future. This commitment to ecological conservation in the context of adaptive learning and management, transparency and inclusive decision-making is indicative of the approach required to pre-empt and avoid social and institutional barriers to large scale restoration and adaptation in the GBR.

### Confronting epistemic and ontological conflict

Though overcoming technical and scientific challenges for adaptation research is critical, it is important that steps are taken sooner rather than later to initiate broad public dialogue over the goals of intervention. Assisted adaptation research is inherently prospective and involves considerable uncertainty over benefits, impacts, and the morality of science and engineering interventions in area as special as the GBR [81]. For example, the exploration of solar radiation management in the GBR could be perceived by the public as a way for the government to justify government inaction on greenhouse gas mitigation; as this could be seen as addressing the symptoms and not the causes of environmental degradation and climate change. Engagement in ecosystem restoration can also unearth past conflicts over short term use versus long-term sustainable management of the marine and catchment environment, most of which are ongoing [82].

Behind the seemingly mundane observation that different goals are likely to lead to different outcomes is the need to consider what kinds of local and regional ecosystems, communities, and resource-based industries may be both desirable and achievable in the GBR in the face of global environmental change. The ideal of objectivity characteristic of scientific knowledge is critical to articulating what is achievable. However, it must be recognized that neither scientists nor management agencies can function solely as disinterested observers or suppliers of facts in the articulation of goals. Assumptions about authenticity and naturalness that underlie notions of ecological value should be transparent to all stakeholders and subject to deliberation.

Histories of engagement and the goals of intervention research can impact on the trust, approval and support that a community may give to the research and deployment of new technology, and this can impact on the willingness to engage in deliberations about assisted ecosystem adaptation in negative and positive ways [83]. Adaptation research that provides for communication and information about interventions and involves processes for engaging diverse communities, stakeholders, scientists, society, and governments in science and deliberative processes will help to address conflicts.

## Conclusion

Challenges impacting on assisted ecosystem adaptation research range from the complex social conditions of impacted communities and governance arrangements affecting decision making to unresolved scientific debates, the highly prospective nature of anticipatory intervention, and the need to confront morally and emotionally difficult questions about the prioritization of ecological and cultural values. It is telling that none of these challenges are likely to be resolved through less engagement with affected communities, stakeholders, diverse scientists, institutional bodies and multiple societies. Achieving an ethical mandate to progress assisted ecosystem research in the GBR and elsewhere depends on genuine processes of participation and negotiation that lead the public, Indigenous communities, scientists, resource users and other stakeholders to perceive decision-making processes as fair and oriented toward social responsibility [84–86].

The complexity of assisted ecosystem adaptation raises challenges for engagement while illustrating the importance of progressing ecological and social research in a transparent, and where possible, participatory manner. In part, this is a matter of understanding how values and beliefs relevant to assisted adaptation differ among stakeholder groups and more effectively communicating the goals and outcomes of adaptation research. It is also a matter of embracing the epistemic and ontological challenges evoked by assisted ecosystem adaptation research in order to promote trust, co-learning and the development of shared goals and decisions.

## Supporting information

S1 Table Types and features of the documents referenced in Table 1. (DOCX)

## References

[1] Keith DW. Geoengineering the climate: History and prospect. Annu Rev Energy Environ. 2000.

[2] Lenton A, Boyd PW, Thatcher M, Emmerson KM. Foresight must guide geoengineering research and development. Nat Clim Chang. 2019.

[3] National Academies of Sciences, Engineering, and Medicine. Reflecting Sunlight—Recommendations for Solar Geoengineering Research and Research Governance. Washington, DC: The National Academies Press.; 2021.

[4] Trisos CH, Merow C, Pigot AL. The projected timing of abrupt ecological disruption from climate change. Nature. 2020;580:496–504. doi: 10.1038/s41586-020-2189-9 32322063

[5] Travis JMJ, Delgado M, Bocedi G, Baguette M, Bartoń K, Bonte D, et al. Dispersal and species’ responses to climate change. Oikos. 2013;122(11):1532–40.

[6] MacCracken M. Impact intervention: Regional geo-engineering as a complementary step to aggressive mitigation. IOP Conference Series Earth and Environmental Science. 2009;6(45).

[7] Aradottir AL, Hagen D. Ecological Restoration: Approaches and Impacts on Vegetation, Soils and Society. Advances in Agronomy. 2013;120:173–222.

[8] Asayama S, Sugiyama M, Ishii A. Ambivalent climate of opinions: Tensions and dilemmas in understanding geoengineering experimentation. Geoforum. 2017;80:82–92.

[9] Rohr JR, Farag AM, Cadotte MW, Clements WH, Smith JR, Ulrich CP, et al. Transforming ecosystems: When, where, and how to restore contaminated sites. Integr Environ Assess Manag. 2016;12(2):273–83. doi: 10.1002/ieam.1668 26033665 PMC4862316

[10] Prober SM, Doerr VAJ, Broadhurst LM, Williams KJ, Dickson F. Shifting the conservation paradigm: a synthesis of options for renovating nature under climate change. Ecological Monographs. 2019;89(1):1–23.

[11] Shi L. From Progressive Cities to Resilient Cities: Lessons from History for New Debates in Equitable Adaptation to Climate Change. Urban Affairs Review. 2020:1–38.

[12] Sansilvestri R, Frascaria-Lacoste N, Fernández-Manjarrés J. One option, two countries, several strategies: subjacent mechanisms of assisted migration implementation in Canada and France. Restoration Ecology. 2016;24(4):489–98.

[13] Heikkila T, Gerlak AK. Investigating Collaborative Processes Over Time. The American Review of Public Administration. 2014;46(2):180–200.

[14] Lockie S, Franetovich M, Sharma S, Rolfe J. Democratisation Versus Engagement? Current Practice in Social Impact Assessment, Economic Impact Assessment and Community Participation in the Coal Mining Industry of the Bowen Basin. Impact Assessment and Project Appraisal. 2008;26(3):177–87.

[15] Marshall NA, Curnock MI, Goldberg J, Gooch M, Marshall PA, Pert PL, et al. The Dependency of People on the Great Barrier Reef, Australia. Coastal Management. 2017;45(6):505–18.

[16] Marshall N, Barnes ML, Birtles A, Brown K, Cinner J, Curnock M, et al. Measuring what matters in the Great Barrier Reef. Front Ecol Environ. 2018;16:271–7.

[17] CoA CoA. Reef Blue Print -Great Barrier Reef Blueprint for Resilience 2017.

[18] GBRMPA GBRMPA. Policy on Great Barrier Reef Interventions. (revision 0/12/2020) 2020.

[19] Ma H, Lv Y, Li H. Complexity of ecological restoration in China. Ecological Engineering. 2013;52:75–8.

[20] Khater C, Raevel V, Sallantin J, Thompson JD, Hamze M, Martin A. Restoring Ecosystems Around the Mediterranean Basin: Beyond the Frontiers of Ecological Science. Restoration Ecology. 2012;20(1):1–6.

[21] Crossman ND, Bryan BA. Identifying cost-effective hotspots for restoring natural capital and enhancing landscape multifunctionality. Ecological Economics. 2009;68(3):654–68.

[22] Mohr JJ, Metcalf EC. The business perspective in ecological restoration: issues and challenges. Restoration Ecology. 2018;26(2):381–90.

[23] Moreno-Cruz JB, Keith DW. Climate policy under uncertainty: a case for solar geoengineering. Climatic Change. 2012;121(3):431–44.

[24] Josephs LI, Humphries AT. Identifying social factors that undermine support for nature-based coastal management. J Environ Manage. 2018;212:32–8. doi: 10.1016/j.jenvman.2018.01.085 29427939

[25] McFadden BR, Lusk JL. Cognitive biases in the assimilation of scientific information on global warming and genetically modified food. Food Policy. 2015;54:35–43.

[26] Hagendijk R, Irwin A. Public Deliberation and Governance: Engaging with Science and Technology in Contemporary Europe. Minerva. 2006;44(2):167–84.

[27] Hoegh-Guldberg O, Hughes L, McIntyre S, Lindenmayer DB, Parmesan C, Possingham HP, et al. Ecology: Assisted colonization and rapid climate change. Science Advances. 2008;321(5887):345–6. doi: 10.1126/science.1157897 18635780

[28] Renn O, Benighaus C. Perception of technological risk: insights from research and lessons for risk communication and management. Journal of Risk Research. 2013;16(3–4):293–313.

[29] Klinke AR, Ortwin. A New Approach to Risk Evaluation and Management: Risk-Based, Precaution-Based, and Discourse-Based Strategies. Risk Analysis. 2002;22(6):1071–94. doi: 10.1111/1539-6924.00274 12530780

[30] Rodríguez-Entrena M, Salazar-Ordóñez M. Influence of scientific-technical literacy on consumers’ behavioural intentions regarding new food. Appetite. 2013;60(1):193–202. doi: 10.1016/j.appet.2012.09.028 23063609

[31] Bruce DM. A Social Contract For Biotechnology: Shared Visions For Risky Technologies? Journal of Agricultural and Environmental Ethics. 2002;15:279–89.

[32] Visser M, Maughan N, Ouled Belgacem A, Neffati M. Stakeholder views on restoring depleted cereal fallows in arid Tunisia: Societal barriers and possible crevices. Journal of Arid Environments. 2011;75(11):1191–200.

[33] Fairbrother M. Geoengineering, moral hazard, and trust in climate science: evidence from a survey experiment in Britain. Climatic Change. 2016;139(3–4):477–89.

[34] Bellamy R, Chilvers J, Vaughan NE, Lenton TM. A review of climate geoengineering appraisals. Wiley Interdisciplinary Reviews: Climate Change. 2012;3(6):597–615.

[35] Merk C, Ponitzsch G. The Role of Affect in Attitude Formation toward New Technologies: The Case of Stratospheric Aerosol Injection. Risk Anal. 2017;37(12):2289–304. doi: 10.1111/risa.12780 28244119

[36] Merk C, Ponitzsch G, Kniebes C, Rehdanz K, Schmidt U. Exploring public perceptions of stratospheric sulfate injection. Climatic Change. 2015;130:299–312.

[37] Burns ET, Flegal JA, Keith DW, Mahajan A, Tingley D, Wagner G. What do people think when they think about solar geoengineering? A review of empirical social science literature, and prospects for future research. Earth’s Future. 2016;4(11):536–42.

[38] Braun C, Merk C, Pönitzsch G, Rehdanz K, Schmidt U. Public perception of climate engineering and carbon capture and storage in Germany: survey evidence. Climate Policy. 2017;18(4):471–84.

[39] Richardson BJ. The Emerging Age of Ecological Restoration Law. Review of European, Comparative & International Environmental Law. 2016;25(3):277–90.

[40] Florin M-VX, J. Risk governance: An overview of drivers and success factors. 2014.

[41] Owen R, Macnaghten P, Stilgoe J. Responsible research and innovation: From science in society to science for society, with society. Science and Public Policy. 2012;39(6):751–60.

[42] Poumadère M, Bertoldo R, Samadi J. Public perceptions and governance of controversial technologies to tackle climate change: nuclear power, carbon capture and storage, wind, and geoengineering. Wiley Interdisciplinary Reviews: Climate Change. 2011;2(5):712–27.

[43] Tuana N, Sriver RL, Svoboda T, Olson R, Irvine PJ, Haqq-Misra J, et al. Towards Integrated Ethical and Scientific Analysis of Geoengineering: A Research Agenda. Ethics, Policy & Environment. 2012;15(2):136–57.

[44] Olsson P, Gunderson LH, Carpenter SR, Ryan P, Lebel L, Folke C, et al. Shooting the Rapids: Navigating Transitions to Adaptive Governance of Social-Ecological Systems. Ecology and Society. 2006;11(1):18.

[45] Gunderson L, Light SS. Adaptive management and adaptive governance in the everglades ecosystem. Policy Sciences. 2006;39(4):323–34.

[46] Kishimoto A. Redefining safety in the era of risk trade-off and sustainability. Journal of Risk Research. 2013;16(3–4):369–77.

[47] Fast S, Nourallah L. Public Trust in Environmental Decision-Making: A Case Study of Shale Gas Regulation in Kent County, New Brunswick. Case Studies in the Environment. 2018:1–7.

[48] Borkhataria RR, Wetzel PR, Henriquez H, Davis SE. The Synthesis of Everglades Restoration and Ecosystem Services (SERES): a case study for interactive knowledge exchange to guide Everglades restoration. Restoration Ecology. 2017;25:S18–S26.

[49] Light AR. Spark Plugs of Policy Implementation: Intergovernmental Relations and Public Participation in Florida’s Acceler8 Initiative to Speed Everglades Restoration. Vermont Law Review. 2006;30:939.

[50] DiEnno CM, Thompson JL. For the love of the land: How emotions motivate volunteerism in ecological restoration. Emotion, Space and Society. 2013;6:63–72.

[51] Burger J. Integrating environmental restoration and ecological restoration: long-term stewardship at the department of energy. Environ Manage. 2000;26(5):469–78. doi: 10.1007/s002670010105 10982725

[52] Sarkar S. Biodiversity and Environmental Philosophy: An Introduction: Cambridge University Press; 2005.

[53] Schwartz MW, Hellmann JJ, McLachlan JM, Sax DF, Borevitz JO, Brennan J, et al. Managed Relocation: Integrating the Scientific, Regulatory, and Ethical Challenges. BioScience. 2012;62(8):732–43.

[54] Corner AP, Nick Like artificial trees? The effect of framing by natural analogy on public perceptions of geoengineering. Climatic Change. 2015;130:425–38.

[55] Corner A, Parkhill K, Pidgeon N, Vaughan NE. Messing with nature? Exploring public perceptions of geoengineering in the UK. Global Environmental Change. 2013;23(5):938–47.

[56] Svoboda T. The Ethics of Geoengineering: Moral Considerability and the Convergence Hypothesis. Journal of Applied Philosophy. 2012;29(3):243–56.

[57] Svoboda T, Irvine P. Ethical and Technical Challenges in Compensating for Harm Due to Solar Radiation Management Geoengineering. Ethics, Policy & Environment. 2014;17(2):157–74.

[58] Corner A, Pidgeon N. Like artificial trees? The effect of framing by natural analogy on public perceptions of geoengineering. Climatic Change. 2015;130:425–38.

[59] Shackley S, McLachlan C, Gough C. The public perception of carbon dioxide capture and storage in the UK: results from focus groups and a survey. Climate Policy. 2011;4(4):377–98.

[60] Koskinen I. Where is the epistemic community? On democratisation of science and social accounts of objectivity. Synthese. 2017;194:4671–86.

[61] Vanclay F. Principles to gain a social licence to operate for green initiatives and biodiversity projects. Current Opinion in Environmental Sustainability. 2017;29:48–56.

[62] Dengler M. Spaces of power for action: Governance of the Everglades Restudy process (1992–2000). Political Geography. 2007;26(4):423–54.

[63] Gerlak AK, Heikkila T. Building a Theory of Learning in Collaboratives: Evidence from the Everglades Restoration Program. Journal of Public Administration Research and Theory. 2011;21(4):619–44.

[64] Kenter JO, Reed MS, Fazey I. The Deliberative Value Formation model. Ecosystem Services. 2016;21:194–207.

[65] Gregory R, Satterfield T, Hasell A. Using decision pathway surveys to inform climate engineering policy choices. PNAS. 2015;113(3):560–5. 10.1073/pnas.1508896113 PMC4725466 26729883

[66] Nanda AVV, Rijke J, Beesley L, Gersonius B, Hipsey MR, Ghadouani A. Matching ecosystem functions with adaptive ecosystem management: Decision pathways to overcome institutional barriers. Water. 2018;10(6).

[67] Agardy T, Claudet J, Day JC. ‘Dangerous Targets’ revisited: Old dangers in new contexts plaguemarine protected areas. Aquatic Conserv: Mar Freshw Ecosyst. 2016;26:7–23.

[68] Patwardhan A, Downing T, Leary N, Wilbanks T. Towards an integrated agenda for adaptation research: theory, practice and policy. Strategy paper. Current Opinion in Environmental Sustainability. 2009;1(2):219–25.

[69] Carrier M. Values and Objectivity in Science: Value-Ladenness, Pluralism and the Epistemic Attitude. Science and Education. 2013;22(10):2547–68.

[70] Hulme MM M. Climate change: What do we know about the IPCC? Progress in Physical Geography. 2010;34(5):705–18.

[71] Baker JT, Furnas M, Johnson A, Moss A, Pearson R, Rayment G, et al. A report on the study of land-sourced pollutants and their impacts on water quality in and adjacent to the Great Barrier Reef: an assessment to guide the development of management plans to halt any decline in the water quality of river catchments draining to the Reef, as a result of land-based pollution, and to achieve the long-term goal of reversing any trend in declining water quality. Brisbane, Australia; 2003.

[72] Shindler BC, Aldred K. Integrating citizens in adaptive management: a propositional analysis. Conservation Ecology. 1999;3(1).

[73] Kates RW, Travis WR, Wilbanks TJ. Transformational adaptation when incremental adaptations to climate change are insufficient. Proc Natl Acad Sci U S A. 2012;109(19):7156–61. doi: 10.1073/pnas.1115521109 22509036 PMC3358899

[74] Burns WCGF J.A. Climate Geoengineering and the Role of Public Deliberation: A Comment on the us National Academy of Sciences’ Recommendations on Public Participation. Climate Law. 2015;5(2–4):252–94.

[75] Dale A, Vella K, Pressey R, Brodie J, Gooch M, Potts R, et al. Risk analysis of the governance system affecting outcomes in the Great Barrier Reef. Journal of Environmental Management. 2016;183(3):712–21. doi: 10.1016/j.jenvman.2016.09.013 27641654

[76] Gruby RL, Gray NJ, Campbell LM, Acton L. Toward a Social Science Research Agenda for Large Marine Protected Areas. Conservation Letters. 2016;9(3):153–63.

[77] Ban NC, Davies TE, Aguilera SE, Brooks C, Cox M, Epstein G, et al. Social and ecological effectiveness of large marine protected areas. Global Environmental Change. 2017;43:82–91.

[78] Mitchell NJ, Rodriguez N, Kuchling G, Arnall SG, Kearney MR. Reptile embryos and climate change: Modelling limits of viability to inform translocation decisions. Biological Conservation. 2016;204:134–47.

[79] Reed MS, Vella S, Challies E, de Vente J, Frewer L, Hohenwallner-Ries D, et al. A theory of participation: what makes stakeholder and public engagement in environmental management work? Restoration Ecology. 2018;26:S7–S17.

[80] Vella K, Baresi U. Understanding How Policy Actors Improvise and Collaborate in the Great Barrier Reef. Coastal Management. 2017;45(6):487–504.

[81] Taylor B, Hussey K, Fidelman P, Vella K, Maclean K, Newlands M, et al. Reef Restoration and Adaptation Program: Engagment and Regulatory Dimensions. A report provided to the Australian Government by the Reef Restoration and Adaptation Program. 2019.

[82] Berardo R, Heikkila T, Gerlak AK. Interorganizational Engagement in Collaborative Environmental Management: Evidence from the South Florida Ecosystem Restoration Task Force. Journal of Public Administration Research and Theory. 2014;24(3):697–719.

[83] Huijts NMA, Molin EJE, Steg L. Psychological factors influencing sustainable energy technology acceptance: A review-based comprehensive framework. Renewable and Sustainable Energy Reviews. 2012;16(1):525–31.

[84] Moffat K, Lacey J, Zhang A, Leipold S. The social licence to operate: a critical review. Forestry. 2015;89(5):477–88.

[85] Lacey J, Edwards P, Lamont J. Social licence as social contract: procedural fairness and forest agreement-making in Australia. Forestry. 2016;89(5):489–99.

[86] Falck WE. Social licencing in mining—between ethical dilemmas and economic risk management. Mineral Economics. 2016;29(2–3):97–104.

