# Supplementary material for: Challenges and opportunities for assisted regional ecosystem adaptation: International experience and implications for adaptation research
Source: PLoS One. 2021 Sep 24;16(9):e0257868. doi: 10.1371/journal.pone.0257868 (PMC8462708; doi:10.1371/journal.pone.0257868)
Supplement: S1 Table — (DOCX) [file pone.0257868.s001.docx]

**S1 Table. Types and features of the documents referenced in Table 1.**

| **Authors** | **Year** | **Document Title** | **Source** | **Fields (SciMagoJR)** | **Nature of document (conceptual, empirical)** |
| --- | --- | --- | --- | --- | --- |
| Aradottir, A. L., & Hagen, D. | 2013 | Ecological Restoration: Approaches and Impacts on Vegetation, Soils and Society | Advances in Agronomy | Agricultural and Biological Sciences | Conceptual |
| Asayama, S., Sugiyama, M., & Ishii, A. | 2017 | Ambivalent climate of opinions: Tensions and dilemmas in understanding geoengineering experimentation | Geoforum | Social Sciences | Empirical (Japan) |
| Bellamy, R., Chilvers, J., Vaughan, N. E., & Lenton, T. M. | 2012 | A review of climate geoengineering appraisals | Wiley Interdisciplinary Reviews: Climate Change | Earth and Planetary Sciences | Conceptual |
|  |  |  |  | Environmental Science |  |
|  |  |  |  | Social Sciences |  |
| Borkhataria, R. R., Wetzel, P. R., Henriquez, H., & Davis, S. E. | 2017 | The Synthesis of Everglades Restoration and Ecosystem Services (SERES): a case study for interactive knowledge exchange to guide Everglades restoration | Restoration Ecology | Agricultural and Biological Sciences | Empirical (United States) |
|  |  |  |  | Environmental Science |  |
| Braun, C., Merk, C., Pönitzsch, G., Rehdanz, K., & Schmidt, U. | 2017 | Public perception of climate engineering and carbon capture and storage in Germany: survey evidence | Climate Policy | Earth and Planetary Sciences | Empirical (Germany) |
|  |  |  |  | Environmental Science |  |
| Bruce, D. M. | 2002 | A social contract for biotechnology: Shared visions for risky technologies? | Journal of Agricultural and Environmental Ethics | Agricultural and Biological Sciences | Empirical (United Kingdom) |
|  |  |  |  | Arts and Humanities |  |
|  |  |  |  | Environmental Science |  |
| Burger, J. | 2000 | Integrating environmental restoration and ecological restoration: long-term stewardship at the department of energy | Environmental Management | Environmental Science | Empirical (United States) |
| Burns, E. T., Flegal, J. A., Keith, D. W., Mahajan, A., Tingley, D., & Wagner, G. | 2016 | What do people think when they think about solar geoengineering? A review of empirical social science literature, and prospects for future research | Earth's Future | Earth and Planetary Sciences | Conceptual |
|  |  |  |  | Environmental Science |  |
| Corner, A., Parkhill, K., Pidgeon, N., & Vaughan, N. E. | 2013 | Messing with nature? Exploring public perceptions of geoengineering in the UK | Global Environmental Change | Environmental Science | Empirical (United Kingdom) |
|  |  |  |  | Social Sciences |  |
| Corner, A. P. & Pidgeon, N. | 2015 | Like artificial trees? The effect of framing by natural analogy on public perceptions of geoengineering | Climatic Change | Earth and Planetary Sciences | Empirical (United Kingdom) |
|  |  |  |  | Environmental Science |  |
| Crossman, N. D., & Bryan, B. A. | 2009 | Identifying cost-effective hotspots for restoring natural capital and enhancing landscape multifunctionality | Ecological Economics | Economics, Econometrics and Finance | Empirical (Australia) |
|  |  |  |  | Environmental Science |  |
| DiEnno, C. M., & Thompson, J. L. | 2013 | For the love of the land: How emotions motivate volunteerism in ecological restoration | Emotion, Space and Society | Psychology | Empirical (United States) |
| Fairbrother, M. | 2016 | Geoengineering, moral hazard, and trust in climate science: evidence from a survey experiment in Britain | Climatic Change | Earth and Planetary Sciences | Empirical (United Kingdom) |
|  |  |  |  | Environmental Science |  |
| Fast, S., & Nourallah, L. | 2018 | Public Trust in Environmental Decision-Making: A Case Study of Shale Gas Regulation in Kent County, New Brunswick | Case Studies in the Environment | Not listed | Empirical (Canada) |
| Florin, M.-V., Xu, J. | 2014 | Risk governance: An overview of drivers and success factors | -Book- | -Book- | Conceptual |
| Gunderson, L., & Light, S. S. | 2006 | Adaptive management and adaptive governance in the Everglades ecosystem | Policy Sciences | Environmental Science | Empirical (United States) |
|  |  |  |  | Social Sciences |  |
| Hagendijk, R., & Irwin, A. | 2006 | Public Deliberation and Governance: Engaging with Science and Technology in Contemporary Europe | Minerva | Social Sciences | Empirical (Europe) |
| Hoegh-Guldberg, O., Hughes, L., McIntyre, S., Lindenmayer, D. B. P., C., Possingham, H.P., & Thomas, C. D. | 2008 | Ecology: Assisted colonization and rapid climate change | Science Advances | Multidisciplinary | Conceptual |
| Josephs, L. I., & Humphries, A. T. | 2018 | Identifying social factors that undermine support for nature-based coastal management | Journal of Environmental Management | Environmental Science | Empirical (United States) |
|  |  |  |  | Medicine |  |
| Khater, C., Raevel, V., Sallantin, J., Thompson, J. D., Hamze, M., & Martin, A. | 2012 | Restoring Ecosystems Around the Mediterranean Basin: Beyond the Frontiers of Ecological Science | Restoration Ecology | Agricultural and Biological Sciences | Empirical (Europe, Africa) |
|  |  |  |  | Environmental Science |  |
| Kishimoto, A. | 2013 | Redefining safety in the era of risk trade-off and sustainability | Journal of Risk Research | Business, Management and Accounting | Conceptual |
|  |  |  |  | Engineering |  |
|  |  |  |  | Social Sciences |  |
| Klinke, A. R., Ortwin. | 2002 | A New Approach to Risk Evaluation and Management: Risk-Based, Precaution-Based, and Discourse-Based Strategies | Risk Analysis | Engineering | Conceptual |
|  |  |  |  | Medicine |  |
| Light, A. R. | 2006 | Spark Plugs of Policy Implementation: Intergovernmental Relations and Public Participation in Florida's Acceler8 Initiative to Speed Everglades Restoration | Vermont Law Review | Not listed | Empirical (United States) |
| Ma, H., Lv, Y., & Li, H. | 2013 | Complexity of ecological restoration in China | Ecological Engineering | Environmental Science | Empirical (China) |
| McFadden, B. R., & Lusk, J. L. | 2015 | Cognitive biases in the assimilation of scientific information on global warming and genetically modified food | Food Policy | Agricultural and Biological Sciences | Conceptual |
|  |  |  |  | Economics, Econometrics and Finance |  |
|  |  |  |  | Environmental Science |  |
|  |  |  |  | Social Sciences |  |
| Merk, C., & Ponitzsch, G. | 2017 | The Role of Affect in Attitude Formation toward New Technologies: The Case of Stratospheric Aerosol Injection | Risk Analysis | Engineering | Empirical (Germany) |
|  |  |  |  | Medicine |  |
| Merk, C. P., Gert Kniebes, Carola, & Rehdanz, K. S., Ulrich. | 2015 | Exploring public perceptions of stratospheric sulfate injection | Climatic Change | Earth and Planetary Sciences | Empirical (Germany) |
|  |  |  |  | Environmental Science |  |
| Mohr, J. J., & Metcalf, E. C. | 2018 | The business perspective in ecological restoration: issues and challenges | Restoration Ecology | Agricultural and Biological Sciences | Empirical (United States) |
|  |  |  |  | Environmental Science |  |
| Moreno-Cruz, J. B., & Keith, D. W. | 2012 | Climate policy under uncertainty: a case for solar geoengineering | Climatic Change | Earth and Planetary Sciences | Conceptual |
|  |  |  |  | Environmental Science |  |
| Olsson, P. G., Lance H., Carpenter, S. R., Ryan, P., Lebel, L., Folke, C., & Holling, C. S. | 2006 | Shooting the Rapids: Navigating Transitions to Adaptive Governance of Social-Ecological Systems | Ecology and Society | Environmental Science | Empirical (United States) |
| Owen, R., Macnaghten, P., & Stilgoe, J. | 2012 | Responsible research and innovation: From science in society to science for society, with society | Science and Public Policy | Environmental Science | Empirical (Europe) |
|  |  |  |  | Social Sciences |  |
| Poumadère, M., Bertoldo, R., & Samadi, J. | 2011 | Public perceptions and governance of controversial technologies to tackle climate change: nuclear power, carbon capture and storage, wind, and geoengineering | Wiley Interdisciplinary Reviews: Climate Change | Earth and Planetary Sciences | Conceptual |
|  |  |  |  | Environmental Science |  |
|  |  |  |  | Social Sciences |  |
| Renn, O., & Benighaus, C. | 2013 | Perception of technological risk: insights from research and lessons for risk communication and management | Journal of Risk Research | Business, Management and Accounting | Conceptual |
|  |  |  |  | Engineering |  |
|  |  |  |  | Social Sciences |  |
| Richardson, B. J. | 2016 | The Emerging Age of Ecological Restoration Law | Review of European, Comparative & International Environmental Law | Environmental Science | Empirical (Europe) |
|  |  |  |  | Social Sciences |  |
| Rodríguez-Entrena, M., & Salazar-Ordóñez, M. | 2013 | Influence of scientific-technical literacy on consumers' behavioural intentions regarding new food | Appetite | Nursing | Conceptual |
|  |  |  |  | Psychology |  |
| Rohr, J. R., Farag, A. M., Cadotte, M. W., Clements, W. H., Smith, J. R., Ulrich, C. P., & Woods, R. | 2016 | Transforming ecosystems: When, where, and how to restore contaminated sites | Integrated Environmental Assessment and Management | Environmental Science | Conceptual |
|  |  |  |  | Medicine |  |
|  |  |  |  | Social Sciences |  |
| Sarkar, S. | 2005 | Biodiversity and Environmental Philosophy: An Introduction | -Book- | -Book- | Conceptual |
| Schwartz, M. W., Hellmann, J. J., McLachlan, J. M., Sax, D. F., Borevitz, J. O., Brennan, J., Camacho, A. E., Ceballos, G., Clark, J. R., Doremus, H., Early, R., Etterson, J. R., Fielder, D., Gill, J. L., Gonzalez, P., Green, N., Hannah, L., Jamieson, D. W., Javeline, D., Minteer, B. A., Odenbaugh, J., Polasky, S., Richardson, D. M., Root, T. L., Safford, H. D., Sala, O., Schneider, S. H., Thompson, A. R., Williams, J. W., Vellend, M., Vitt, P., & Zellmer, S. | 2012 | Managed Relocation: Integrating the Scientific, Regulatory, and Ethical Challenges | BioScience | Agricultural and Biological Sciences | Empirical (United States) |
| Svoboda, T. | 2012 | The Ethics of Geoengineering: Moral Considerability and the Convergence Hypothesis | Journal of Applied Philosophy | Arts and Humanities | Conceptual |
| Svoboda, T., & Irvine, P. | 2014 | Ethical and Technical Challenges in Compensating for Harm Due to Solar Radiation Management Geoengineering | Ethics, Policy & Environment | Arts and Humanities | Conceptual |
|  |  |  |  | Environmental Science |  |
|  |  |  |  | Social Sciences |  |
| Tuana, N., Sriver, R. L., Svoboda, T., Olson, R., Irvine, P. J., Haqq-Misra, J., & Keller, K. | 2012 | Towards Integrated Ethical and Scientific Analysis of Geoengineering: A Research Agenda | Ethics, Policy & Environment | Arts and Humanities | Conceptual |
|  |  |  |  | Environmental Science |  |
|  |  |  |  | Social Sciences |  |
| Visser, M., Maughan, N., Ouled Belgacem, A., & Neffati, M. | 2011 | Stakeholder views on restoring depleted cereal fallows in arid Tunisia: Societal barriers and possible crevices | Journal of Arid Environments | Agricultural and Biological Sciences | Empirical (Tunisia) |
|  |  |  |  | Earth and Planetary Sciences |  |
|  |  |  |  | Environmental Science |  |
